# ECG autoencoder based on low-rank attention

**DOI:** 10.1038/s41598-024-63378-0

**Published:** 2024-06-04

**Authors:** Shilin Zhang, Yixian Fang, Yuwei Ren

**Affiliations:** 1 School of Information Science and Engineering (Institute of Data Science and Technology), Shandong Normal University, Jinan, 250014 China; 2 School of Information Engineering, Shandong Management University, Jinan, 250357 China

**Keywords:** Cardiovascular diseases, Computer science

## Abstract

The prevalence of cardiovascular disease (CVD) has surged in recent years, making it the foremost cause of mortality among humans. The Electrocardiogram (ECG), being one of the pivotal diagnostic tools for cardiovascular diseases, is increasingly gaining prominence in the field of machine learning. However, prevailing neural network models frequently disregard the spatial dimension features inherent in ECG signals. In this paper, we propose an ECG autoencoder network architecture incorporating low-rank attention (LRA-autoencoder). It is designed to capture potential spatial features of ECG signals by interpreting the signals from a spatial perspective and extracting correlations between different signal points. Additionally, the low-rank attention block (LRA-block) obtains spatial features of electrocardiogram signals through singular value decomposition, and then assigns these spatial features as weights to the electrocardiogram signals, thereby enhancing the differentiation of features among different categories. Finally, we utilize the ResNet-18 network classifier to assess the performance of the LRA-autoencoder on both the MIT-BIH Arrhythmia and PhysioNet Challenge 2017 datasets. The experimental results reveal that the proposed method demonstrates superior classification performance. The mean accuracy on the MIT-BIH Arrhythmia dataset is as high as 0.997, and the mean accuracy and $F_1$-score on the PhysioNet Challenge 2017 dataset are 0.850 and 0.843.

## Introduction

According to the World Health Organization (WHO) report, cardiovascular diseases (CVD) have emerged as the ”number one killer”, posing a significant threat to human health and accounting for up to one-third of all deaths^1^. To address the shortage of cardiologists and medical equipment^2^, the urgent need for pre-screening of suspected patients has arisen^3^. In recent years, propelled by intelligent technology and the internet of things, numerous automatic identification and analysis technologies for electrocardiograms (ECG)^4^ have emerged, marking a new research hotspot in the field of intelligent healthcare.

The detection and recognition of ECG signals carry substantial clinical significance, given the complexity and variability of cardiovascular diseases, which often require diagnosis by experienced physicians. However, the scarcity of experienced doctors or experts, combined with the challenging task of analyzing a large number of ECG recordings and the potential for diagnostic errors due to physician fatigue, underscores the necessity of computer-aided early diagnosis in clinical cardiovascular disease management as a prevailing trend. Simultaneously, to achieve improved results, contemporary machine learning networks often involve a substantial number of layers and learning parameters, resulting in low learning efficiency of the model and hindering direct application in a clinical setting. Addressing this challenge necessitates the development of an efficient feature extraction strategy for ECG signals.

In this paper, we proposed a low-rank attention autoencoder architecture incorporating low-rank attention, which is specifically designed to efficiently grasping spatial feature, enhance feature extraction accuracy, and significantly improve downstream tasks associated with ECG signals. By comprehending the spatial correlation among distinct points of the ECG signal, abnormalities in the ECG signal essentially give rise to multidimensional alterations, and capturing these spatial variations can precisely extract potential signal characteristics, ultimately enhancing the accuracy of cardiovascular disease detection and diagnosis. The main contribution of this paper can be summarized as twofold:

1. It offers a novel perspective for comprehending the correlation between ECG signal dimensions and spatial dimensions, effectively extracting the spatial dimension features of ECG signals, and seamlessly integrating them into the domain of ECG signal classification applications.
2. By incorporating the correlation between dimensions of the ECG signal into an autoencoder architecture and simultaneously optimizing data dimensionality, LRA-autoencoder model demonstrates superior performance compared to other pure autoencoder models based on experimental results.

## Related work

In recent years, there has been a significant surge in the application of deep learning across interdisciplinary fields, with a pronounced focus on its utilization in medical imaging. Simultaneously, there is noteworthy emphasis on the continual advancement of classification algorithms specifically designed for analyzing ECG signals. Deep learning demonstrates the ability to autonomously extract relevant features from data, eliminating the need for manual feature extraction by machine algorithms and thereby reducing potential human error that could affect classification accuracy. Convolutional Neural Networks (CNNs) and autoencoder networks have shown robust performance in ECG signal classification. Prominent CNN models such as VGG, AlexNet, and ResNet-18 have been widely employed for this purpose^5^.

Currently, the end-to-end model is widely adopted by researchers as it enables direct mapping of raw data to classification results. Singh, P et al.^6^ proposes attention-based convolutional denoising autoencoder (ACDAE) effectively denoises the low SNR ECG signal while integrating channel attention. The method combines sparse representation with neural networks to propose an interpretable denoising network. Simultaneously, a weight allocation module is designed to enhance the efficiency of hyperparameter selection. This network exhibits excellent interpretability^7^. The autoencoder network has garnered significant attention from researchers in the field of ECG classification and detection, with the improved model based on this network being widely utilized for ECG classification tasks^8^.

With the remarkable performance of transformer in natural language processing, attention mechanisms have garnered extensive attention from researchers and have been applied across multiple domains. The paper introduces an automated ECG classification method that combines convolutional and attention mechanisms, referred to as the Non-Local Convolutional Block Attention Module (NCBAM)^9^. Jing Zhang et al.^10^ combines convolutional recurrent networks with attention mechanisms to more effectively capture the temporal features of electrocardiogram signals. Sajad Mousavi et al.^11^ proposes a hybrid model named HAN-ECG, which integrates three levels of attention (Wave attention, Beat Attention, and Window Attention) with RNN for AF detection, showcasing promising performance. Tianqi Fan et al.^12^ introduces a network architecture called Convolution Block Attention Module (CRAM), which integrates deep neural networks with channel attention. Most existing networks employ attention mechanisms to highlight the temporal and morphological features of electrocardiogram (ECG) signals, but often overlook the spatial characteristics within ECG signals and inter-signal features. This paper introduces a low-rank attention mechanism that prioritizes spatial features, enriches inter-class diversity, and enhances the accuracy of cardiovascular disease detection.

## Materials and methods

**Figure 1:**
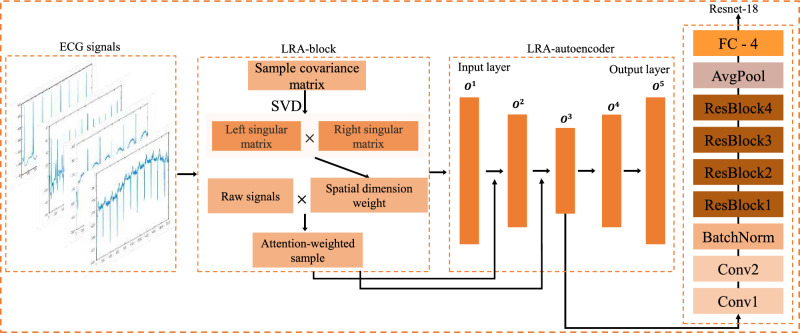
Model structure.

In this paper, we propose an innovative model for ECG classification, illustrated in Fig. 1, which consists of three distinct stages. The LRA-block is utilized to extract spatial features from ECG signals, which are then used as weights to emphasize the class-specific characteristics of the signals.

### ResNet-18

Residual networks have exhibited substantial potential in the domain of image classification, with their fundamental residual concept offering researchers a novel strategy to tackle the challenge of network performance degradation with increasing layer depth. The incorporation of this residual concept into neural networks has produced impressive results, prompting researchers to extend its application to the field of ECG signal analysis as shown in Fig. 1.

**Figure 2:**
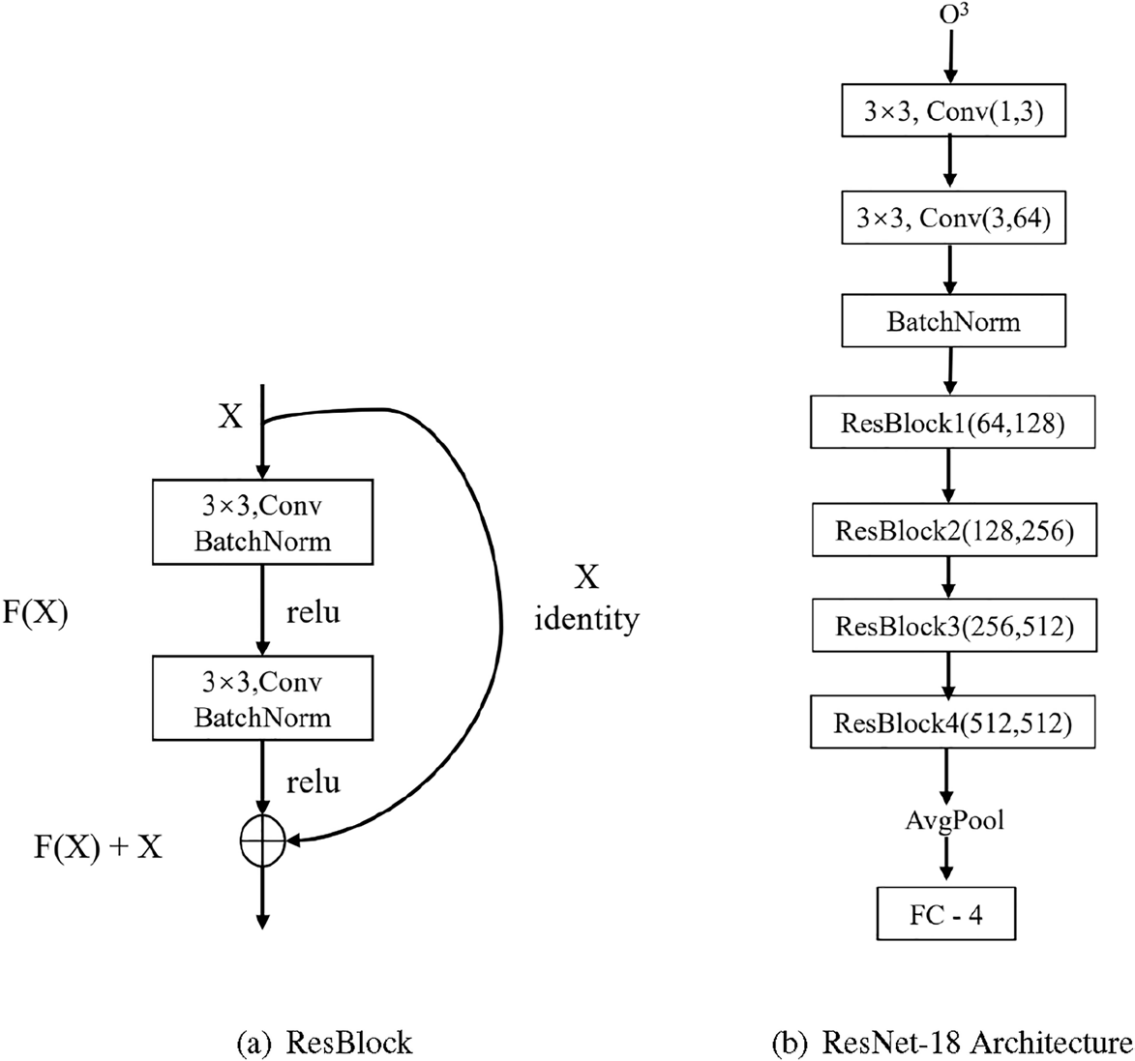
ResBlock and ResNet-18 Architecture.

In this paper, the residual concept is utilized along with the lightest version of ResNet-18 in the ResNet network as the classifier. Through experimentation, it was discovered that ResNet-18 has fewer parameters, thereby reducing the hardware requirements for experiments. Moreover, the overall accuracy of cardiovascular disease detection is on par with other deep models, yet ResNet-18 demonstrates notably enhanced training efficiency. For example, the decision not to choose the recent and popular transformer model was based on the observation that, under equivalent accuracy conditions, ResNet-18 boasts fewer network parameters, higher learning efficiency, requires less demanding training conditions, and is more readily applicable in clinical settings. However, since ResNet was originally designed for the 2D image classification task, we made some changes for the 1D ECG signal detection task. The parameters of the ResNet-18 framework used in this paper are shown in the Fig. 2b. Since ECG is a one dimensional signal, the input in this paper consists of one channel. Subsequently, this single channel is transformed into three channels through a convolution operation for further processing.

### Proposed network architecture

This paper introduces a network that incorporates low-rank attention based on an autoencoder to enhance the categorization of ECG signals. As illustrated in Fig. 1, we integrated the low-rank attention mechanism to facilitate attentive weighting on our data, thereby augmenting the model’s ability to capture salient information within the input. The process initiates with the incorporation of low-rank attentive data, acquired through the low-rank attention block, into the autoencoder network. The resulting features, indicative of the spatially-attentive characteristics, are subsequently transmitted to the ResNet-18 network for the final classification stage.

#### Low-rank attention block

The self-attention mechanism is employed to process sequential data, wherein each element in the sequence is assigned a weight to measure its correlation with other elements, resulting in a comprehensive and detailed correlation. In contrast to traditional RNNs that solely consider the relationship between adjacent elements, self-attention offers a more holistic and intricate approach. We first review the self-attention mechanism^13^. Given an input features $X\in {R}^{n\times d}$, where *n* is the number of samples and *d* is the number of feature dimensions. The self-attention mechanism recodes the input into a query matrix $Q\in {R}^{n\times d'}$, a key matrix $K\in {R}^{n\times d'}$ and a value matrix $V\in {R}^{n\times d}$ through three trainable weights $W^Q$, $W^K$, $W^V$. The attention coefficient *A* can be calculated as:

1 $$\begin{aligned}{} & {} \begin{aligned}{}&\left\{ \begin{array}{lr} Q=W^QX, K= W^KX, V=W^VX;&{} \\ A=(a_{ij})= softmax(\frac{Qk^{T}}{\sqrt{d_{k} } } );&{} \\ X_{out}=AV. &{} \end{array} \right. \end{aligned}{} & {} \end{aligned}$$

where *A* is the attention matrix and $a_{ij}$ indicates the similarity between the *i*-th element and the *j*-th element.The dimension of vector $d_k$ serves as both a query and key.

When dealing with one dimensional data, the dimensions of both the query and key vectors are set to one. The simplified version of self-attention allows for calculating attention directly using the input features *X*:

2 $$\begin{aligned}{} & {} \begin{aligned}{}&\left\{ \begin{array}{lr} A=(a_{ij})= softmax(XX^T);&{} \\ X_{out}=AX. &{} \end{array} \right. \end{aligned}{} & {} \end{aligned}$$

Here, the attention is acquired by computing the similarity among all samples within the feature space.

However, the high computational complexity $O(n^2d)$ of self-attention remains a significant drawback for large sample size datasets, even with simplification. Moreover, this attention mechanism solely captures inter-sample similarity and fails to consider the similarity between feature attributes, hampers its overall capability and flexibility. Notably, differences in attribute dimensions play a crucial role in classification detection. In light of these limitations, we propose calculating attention based on the properties across different samples.

The sample covariance is widely recognized as a measure of the correlation between feature dimensions in a sample. Considering that the left singular vector in SVD represents the frequency of correlation between feature dimensions, and the right singular vector represents the occurrence count of each key dimension, we utilize the product of these vectors to capture the correlation weights effectively, thereby highlighting differences between dimensions. This product is then standardized as dimension attention weight to enhance dissimilarity among original sample dimensions, facilitating subsequent algorithmic classification. The external law-rank attention can be formulated as:

3 $$\begin{aligned}{} & {} \begin{aligned} F(X) =&\left\{ \begin{array}{lr} (U,S,V)=svd\left( X^T X\right) ;&{} \\ A=(a_{ij})=Norm(UV);&{} \end{array} \right. \end{aligned}{} & {} \end{aligned}$$

where *X* is the original data, $X^T$ is the transpose of *X*, *U* is the left singular value matrix and *V* is the right singular value matrix, *svd* expresses singular value decomposition.

The attention calculation in this case involves the multiplication of left and right singular matrices obtained from matrix singular value decomposition. Therefore, softmax normalization is not suitable for this type of attention, and thus we employ the double normalization method^14^. Therefore, the *Norm*() function in Eq. 3 is defined as:

4 $$\begin{aligned}{} & {} \begin{aligned}{}&\left\{ \begin{array}{lr} \bar{a}_{ij} = (UV)_{ij} ;&{} \\ \tilde{a}_{ij}=\frac{exp(\bar{a}_{ij} )}{\sum \limits _iexp(\bar{a}_{ij} )};&{} \\ a_{ij}=\frac{\tilde{a}_{ij}}{\sum \limits _j\tilde{a}_{ij}}. &{} \end{array} \right. \end{aligned}{} & {} \end{aligned}$$

The LRA-block proposed in this paper combines the low-rank attention mechanism with singular value decomposition and modifies the norm function to capture spatial features between dimensions of ECG signals. Utilizing this spatial information as weights enhances the original data to incorporate more informative content. As a result, the reconstructed data can better capture the correlations among the same data in different dimensions and among different data. The LRA-block assumes a critical role in extracting spatial potential features across multiple dimensions of the ECG signal. Their features are subsequently combined to augment signal representation. The spatial potential characteristics of the final ECG signal can be derived by computing the inner product between the left and right singular value matrices. By scrutinizing the intrinsic spatial characteristics of the ECG signal, a more thorough comprehension of its intricate features can be attained. Consequently, this process facilitates an improved extraction of features for subsequent tasks, including classification and detection.

#### Low-rank attention autoencoder

The autoencoder model employs an encoder structure to diminish the dimensionality of ECG signals, eliminate noise and non-significant features, and facilitate feature extraction while concurrently reducing model parameters for accelerated convergence. The raw data passes through the LRA-block to obtain the weight F(X) as shown in Eq. 3, which is then assigned to the data as input into the autoencoder. As depicted in the figure, the output of each layer of the autoencoder’s encoder undergoes the LRA-block operation to acquire the corresponding weight, which is subsequently added to the original output as input for the next layer. The encoder of the autoencoder facilitates data compression and reduction of data dimensions. Following dimensionality reduction, data of varying dimensions contain slightly different information; hence, we incorporate the LRA-block to the output of each encoder layer to obtain the weights for different dimensions. Evidently, this approach enables more comprehensive extraction of spatial features from the data. The training process of ECG signals in the network and the parameters used are shown in Eq. 5. The tanh activation function $Tanh(x)=\frac{e^{x}-e^{-x}}{e^{x}+e^{-x}}$ is capable of mapping the entire real number interval to the range of $(-1,1)$.

5 $$\begin{aligned}{} & {} \begin{aligned}{}&\left\{ \begin{array}{lr} O^{1} = Tanh(W_1 (XF(X)) + b_1);&{} \\ O^{2} = Tanh(W_2(O^{1}F(O^{1})) + b_2);&{} \\ O^{3} = Tanh(W_3(O^{2}F(O^{2})) + b_3);&{} \\ O^{3} = Tanh(W_3(O^{2}F(O^{2})) + b_3);&{} \\ O^{5} =Tanh(W_5(Tanh(W_4 O^{3} + b_4) + b_5). &{} \end{array} \right. \end{aligned}{} & {} \end{aligned}$$

where *X* represent the output of the original data subsequent to the application of the LRA-block, $O^{n}$ is the output of LRA-autoencoder layer ($n = (1, 2, 3, 4, 5)$), $W_i$ represents the weights of layer i in the network, while $b_i$ represents the biases of layer i in the network ($i = (1, 2, 3,4)$).

In this study, the LRA-block is utilized to extract the spatial dimension features inherent in ECG signals and subsequently allocate them as weights to the signals. The principal objective of this approach is to bestow upon the ECG space feature, consequently bolstering the precision of classification tasks. In the LRA-block operation, we deliberately choose not to involve the resulting weights in the gradient updating (*F*(*X*), $F(O^{1})$ and $F(O^{2})$) and backpropagation process, but rather to attach them as weights to the ECG signal. The advantage of this decision is that we only allow the extracted spatial features to influence the representation of the ECG signal, without altering the spatial feature information obtained. By setting the gradient’s requires$\_$ grad attribute to false, we ensure that for the weights in the LRA-block, the gradient is not updated, but only exists as a weight assignment. During the network training process, we emphasize the extraction and utilization of spatial features while avoiding the error propagation of weights, thus maintaining stability throughout the network. This approach not only enables our model to concentrate more on learning categorical features but also ensures the purity of spatial features, thereby improving the overall performance of the classification task.

$O^{3}$ is the output of the encoder after LRA-autoencoder. Noise in complex ECG signals can be eliminated and important features for downstream tasks can be captured by changing the data dimensions with the autoencoder. As an input to the ResNet-18 network, $O^{3}$ passes through two convolution layers(Conv1,Conv2), BachNorm, four ResBlocks(ResBlock[i], i = (1, 2, 3, 4), and average pooling(AvgPool) (Eq. 6). Finally, it enters the ECG signal classification via the fully connected layer.

6 $$\begin{aligned}{} & {} \begin{aligned}{}&\left\{ \begin{array}{lr} f_{1} =BatchNorm(Conv2(Conv1(O^{3}));&{} \\ f_{2} =ResBlock2(ResBlock1(f_{1}));&{} \\ f_{3} =FC(AvgPool(ResBlock4(ResBlock3(f_{2}))). &{} \end{array} \right. \end{aligned}{} & {} \end{aligned}$$

where $f_{1}$ is the distilled feature by applying two convolution layers and batchnorm with $O^{3}$ as input, $f_{2}$ is the output result obtained after two ResBlocks, with $f_{1}$ as input. and $f_{3}$ is the four category prediction probabilities obtained after an two ResBlocks, average pooling layer, and finally a fully connected layer with $f_{2}$ as input.

We focus on the interrelationship between various ECG signal points, approach it from a spatial perspective, and utilize the changes induced by abnormal signals in dimensions and inter-dimensions as potential spatial features. Subsequently, we overlay the features obtained from time series to accomplish ECG signal classification. The LRA-autoencoder network is capable of classifying ECG signals based on both spatial and temporal information, thereby providing a comprehensive understanding of the signal’s characteristics. As a result, the LRA-autoencoder network exhibits superior classification performance.

The proposed LRA-autoencoder network model captures the interconnections between different ECG signal points by extracting their spatial dimensional characteristics. The important spatial dimensional features obtained through the LRA-block are utilized as weights to reconstruct the features of the encoder’s output, which in turn serve as the input to the next layer of the encoder. In this paper, the proposed LRA-autoencoder applies weighted LRA-block processing to the original data and the output of each layer of the encoder. This is aimed at more comprehensively extracting spatial characteristics of electrocardiogram signals across different dimensions to capture variations among different categories. Therefore, this paper provides a more comprehensive understanding of ECG signals in the spatial dimension, which facilitates the classification of ECG signals and enables convenient clinical application for doctors to aid in diagnosis.

## Experiments

### Dataset

In this study, the proposed algorithm underwent training and validation utilizing two datasets: the MIT-BIH Arrhythmia database (MIT-BIH)^15^ and the PhysioNet Challenge 2017 dataset (PhysioNet 2017)^16^.

The MIT-BIH database encompasses approximately 4,000 long-term electrocardiogram records, with 60% representing inpatient data and 40% outpatient data. Each record spans a sampling duration of 30 minutes, with a frequency of 360 Hz, and is recorded in the two leads as shown in Table 1. It encompasses four types of cardiac rhythms: normal sinus rhythm (N), left bundle branch block (L), right bundle branch block (R), and premature ventricular contractions (V). These specific rhythm types were chosen for model training and evaluation.

**Table 1:** Summary of MIT-BIH and PhysioNet 2017, including records sampling rate and number of leads.

Datasets	Records (length)	Sample rate	Number of leads
MIT-BIH	30min	360Hz	2
PhysioNet 2017	30s	300Hz	1

The PhysioNet 2017 dataset constitutes a classification set of single-lead ECG recordings designed for the identification of atrial fibrillation. The recordings have a sampling time of 30 seconds and a sampling frequency of 300 Hz. This dataset categorizes ECG recordings into distinct classes, including normal sinus rhythm (N), atrial fibrillation (AF), other cardiac rhythm (Other), and Noisy recordings.

### Data pre-processing

Normalization has been systematically applied to both datasets with the objective of expediting convergence and improving the speed and accuracy of the analysis. The chosen approach employs Z-score standardization (Eq. 7), wherein the data is normalized by computing the mean and standard deviation of the original ECG signal records. Consequently, the processed data manifests a normal distribution, as exemplified in the formula.

7 $$\begin{aligned} Z=\left( x-\mu \right) /\sigma . \end{aligned}$$

where *x* is the recorded single sampling point, $\mu$ is the calculated mean, and $\sigma$ is the calculated standard deviation.

### Experiments environment

The programming environments employed for the training and testing phases in this experiment include Python 3.7, conda 4.5.11, and torch 1.13.0. The experimental setup comprises hardware with 32.0GB of memory, a GPU (NVIDIA GeForce GTX 1080 Ti), and a CPU (Intel Core i7-8700 CPU@3.20GHz).

### Train strategy

The LRA-autoencoder network employs the adam optimizer, effectively regulating the learning rate within a specific range to ensure consistent parameter values throughout the network. During the training of the LRA-autoencoder network for ECG signal feature extraction, we utilize the Mean Squared Error (MSE) loss function, i.e., $MSE = \frac{1}{M} {\textstyle \sum _{i=1}^{M}}(y_{i} - \hat{y}_{i})^{2}$, where $y_{i}$ is the true category and $\hat{y}_{i}$ is the predicted category. We employ the tanh activation function in the network to enhance the transmission of error signals during backpropagation and mitigate the issue of vanishing gradients. Set kernel$\_$size=3, stride=3, padding=0 for the first two convolution layers, kernel$\_$size=3, stride=1, padding=1 for the last three resblocks, passing through a batchnorm layer, as shown in the Fig. 2b, *F*(*X*) is *X* after Conv1 -> BatchNorm -> relu -> Conv2 -> BatchNorm -> relu (Eq. 8), and the final output of ResBlock is $F(X) + X$. Subsequently, the ECG signal classification is achieved by means of an average pooling layer and a fully connected layer.

8 $$\begin{aligned} f(x)=\left\{ \begin{aligned} x \qquad&\ if \ x>0 \\ 0 \qquad&\ if \ x\le 0 \\ \end{aligned} \right. \end{aligned}$$

### Evaluation criteria

We perform a thorough assessment of the complete test set to substantiate the model’s superiority. By scrutinizing the disparity between the predicted and actual categories, four key parameters are derived: true positives (TP), false positives (FP), true negatives (TN), and false negatives (FN). Our model yields crucial evaluation metrics as follows: Precision(P), Recall(R) as Eq. 9, $F_1$ - score as Eq. 10, Accuracy(Acc) as Eq. 11.

9 $$\begin{aligned}{} & {} \quad P=\frac{TP}{TP+FP},\,R=\frac{TP}{TP+FN}. \end{aligned}$$

10 $$\begin{aligned}{} & {} \quad F_{1}-score=\frac{2\ Precision\ Recall}{Precision+Recall}. \end{aligned}$$

11 $$\begin{aligned}{} & {} \quad Acc=\frac{TP+TN}{TP+FN+FP+TN}. \end{aligned}$$

## Results

**Figure 3:**
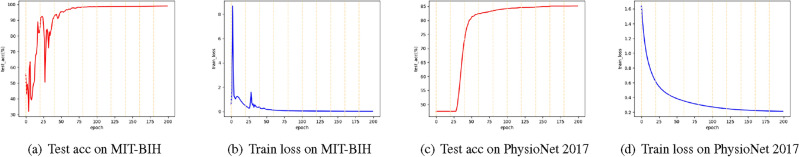
Test acc and train loss on MIT-BIH and PhysioNet 2017.

In our experiment, we selected and utilized the training model with the highest overall classification accuracy for evaluating the testsets. The training and validation processes were conducted on two datasets, namely MIT-BIH and PhysioNet 2017. Figure 3 shows the test accuracy and training loss of the model on the MIT-BIH dataset. It is evident that the model converges after 200 iterations, ultimately achieving minimal training loss and stable test accuracy. Figure 3 demonstrates the training losses and test accuracy of the model during training and testing in the PhysioNet 2017 dataset. The experimental results indicate that our proposed the LRA-autoencoder can effectively achieve the classification and recognition of ECG signals with law-rank attention, rendering it more suitable for clinical applications to assist doctors in diagnosis.

To substantiate the validity of our proposed model, we conducted a comparative analysis with methods utilizing the MIT-BIH and PhysioNet 2017 datasets. The accuracy of our model on the MIT-BIH dataset reaches 0.997, while Recall and $F_1$-score also achieve scores of 0.998 and 0.997, as depicted in Table 2. Notably, the overall accuracy of normal heart rate is particularly significant. Concurrently, we conducted validation on the dataset, and the results are presented in Table 2. The outcomes demonstrate that the proposed LRA-autoencoder network excels in detecting AF when classifying ECG.

**Table 2:** The detailed results in MIT-BIH Arrhythmia and PhysioNet Challenge 2017 datasets.

	Dataset MIT-BIH Arrhythmia				Dataset PhysioNet Chanllenge 2017			
Categories	N	L	R	V	N	AF	Other	Noisy
*Precision*	0.997	0.997	0.997	0.996	0.879	0.902	0.803	1.000
*Recall*	0.998	0.995	0.995	0.996	0.951	0.485	0.860	0.023
$F_1$	0.997	0.996	0.996	0.996	0.913	0.631	0.830	0.046
*Acc*	0.997				0.850			

## Discussion

**Figure 4:**
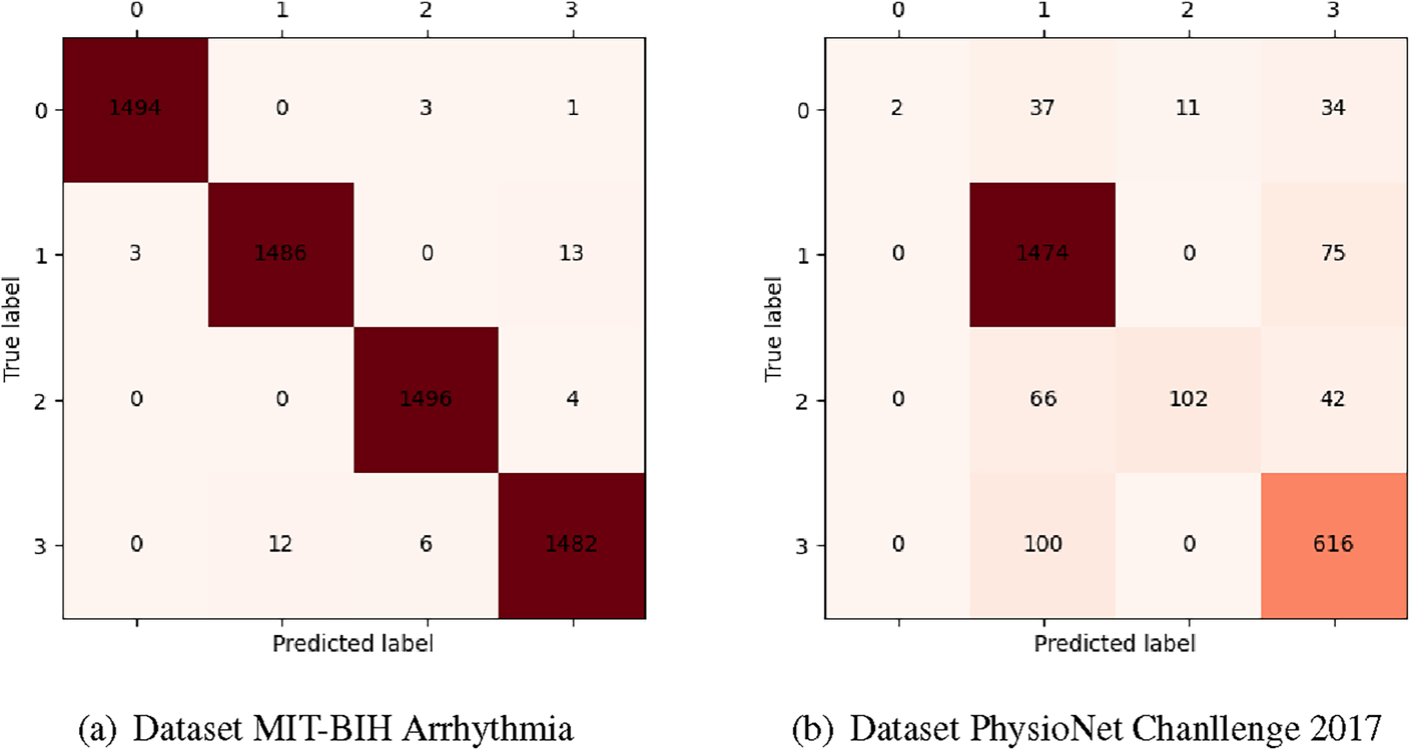
Confusion matrix..

The results of comparing our proposed method with the aforementioned approaches^17,19–24^ on the MIT-BIH dataset are presented in Table 3, and for the PhysioNet 2017 dataset, the results are shown in Table 4. It is evident that the proposed method outperforms other methods, whether on the MIT-BIH dataset or PhysioNet 2017 dataset. Many of these methods utilize neural network architectures such as CNN, LSTM, and DNN. Our proposed LRA-autoencoder method achieves excellent results. It can be seen that the accuracy of the four categories of the MIT-BIH dataset, N, L, R, and V can achieve good results from the confusion matrix shown in Fig. 4a. From Fig. 4a, it is clear that there is a difference between true category and LRA-autoenenencoder predicted category. The amount of data for all categories of MIT-BIH and PhysioNet 2017 datasets are shown in Table 5. The number of four categories in the MIIT-BIH dataset is around 2500, and there is no data imbalance problem, which is conducive to the model for learning. Fig. 4b shows the classification of each category on the 2017 dataset. In Fig. 4b, 0, 1, 2 and 3 correspond to Noisy, N, AF and Other categories. It can be seen that the LRA-autoencoder model proposed in this paper has the best performance in detecting normal ECG signals, followed by other types of cardiovascular diseases, and the worst performance is noisy signals.Through discussion this paper argues that the main reason is that the amount of noise in ECG signal data is less, the lack of enough data to study the modeling, as the amount of data to reduce the accuracy decreases. At the same time, PhysioNet 2017 datasets is also accompanied by the characteristics of data imbalance, and the amount of data between each category is quite different, which brings certain difficulties to the detection of cardiovascular disease.

**Table 3:** The comparision of Acc for others models in MIT-BIH Arrhythmia dataset.

Literatures	Method	Acc
Hou et al^17^	Two-stream Network	0.993
Guo et al^18^	–	0.996
Javid Farhadi et al^19^	Stacked Auto Encoders	0.955
Aryan Odugoudar et al^20^	CNN	0.978
Guang Jun Nicholas Ang et al^21^	YOLOv8n	0.989
Md Shofiqul Islam et al^22^	HARDC	0.990
Che Liu et al^23^	SCDNN	0.990
Negin Alamatsaz et al^24^	1D-CNN+LSTM	0.982
Md Rabiul Islam et al^25^	Convolution+Attention+Transformer	0.995
Muhamad Akbar et al^26^	1D-CNN	0.992
Jutao Wang et al^27^	CNN	0.991
Ours	LRA-autoencoder	**0.997**

**Table 4:** Compare of Acc and $F_1$-score to other models in the PhysioNet Challenge 2017 dataset.

Literatures	Application	Method	$F_1$-score	Acc
Dighanchal Banerjee et al^28^	AF classification	SNN	–	0.770
Wang et al^29^	AF detection	DPRNN	0.829	0.845
Christopher Snyder et al^16^	AF classification	DNN	–	0.740
Fayyazifar^30^	AF detection	NAS	0.824	0.842
Chen et al^31^	AF detection	XGBoost	0.805	0.838
Zihlmann et al^32^	AF classification	CRNN	0.746	0.792
Aoxiang Zhang et al^33^	AF classification	RANet	0.817	-
Jia Xie et al^34^	AF classification	Bi-LSTMAttns	0.823	0.844
Yongyong Chen et al^35^	AF detection	QRS detection	-	0.846
Ours	AF classification	LRA-autoencoder	** 0.843**	** 0.850**

**Table 5:** The amount of data for all categories of MIT-BIH and PhysioNet 2017 datasets.

Data	Categories			
MIT-BIH	N	L	R	V
	2458	2542	2503	2497
PhysioNet 2017	N	AF	Other	Noisy
	1549	210	716	84

**Figure 5:**
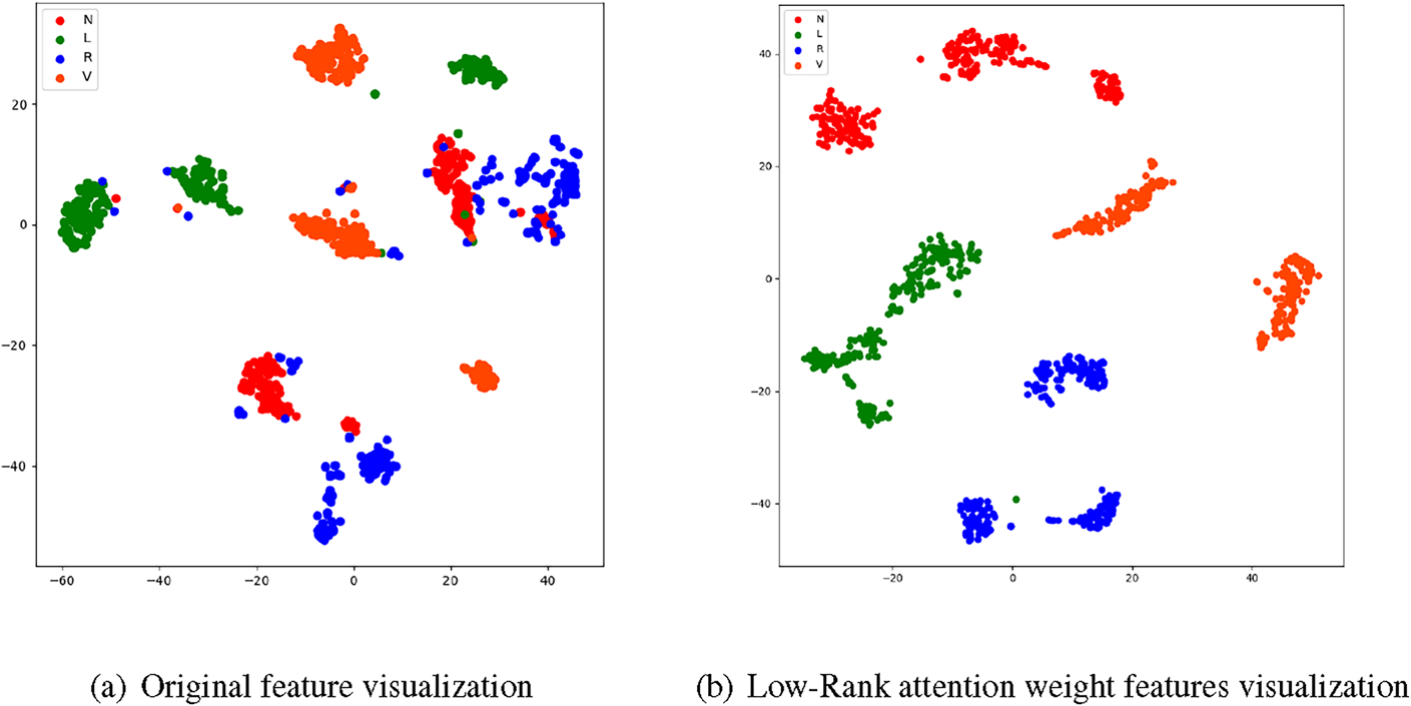
The original feature and with Low-Rank attention weight features visualization.

Figure 5 displays the visualization of both the original data and the ECG signal data with low-rank attention weight features extracted from MIT-BIH. The original data exhibits highly disordered scattering patterns across all four types, lacking discernible regularity. In contrast, employing LRA-autoencoder network to extract features results in the clear categorization of scatter patterns into their respective groups, displaying consistent discharge behavior. This highlights the efficacy of our low-rank attention-based feature extraction approach, which adeptly captures the low-rank attention weight features of ECG signals and facilitates downstream ECG signal processing tasks.

## Conclusion

In this paper, we propose an LRA-autoencoder network specifically designed for ECG signal classification. The LRA-autoencoder emphasizes the spatial dimension features of electrocardiogram signals by introducing an improved Low-rank attention method for extracting these features. Concurrently, it integrates the extracted spatial features with an autoencoder network for dimension optimization, thereby constituting the LRA-autoencoder network. Our approach underwent evaluation on both the MIT-BIH Arrhythmia and PhysioNet Challenge 2017 datasets, with experimental results showcasing the superior performance of this network in ECG classification compared to other methods proposed in this study. However, the LRA-autoencoder network did not demonstrate the same robust classification performance on the imbalanced class PhysioNet Challenge 2017 dataset as it did on the balanced class MIT-BIH Arrhythmia dataset. Thus, there are limitations in handling imbalanced classes with fewer samples.

In the future, there is an increased emphasis on comprehending the spatial characteristics of ECG signals and integrating them into potential features for downstream tasks. This enables accurate identification of classes with fewer samples even in cases of class imbalance. Our future work will primarily revolve around an in-depth exploration of the spatial characteristics inherent in ECG signals.

## Data Availability

Te datasets used and/or analysed during the current study available from the corresponding author on reasonable request.

## References

[1] Liu X, Wang H, Li Z, Qin L (2021). Deep learning in ECG diagnosis: A review. Knowl.-Based Syst..

[2] Gouda P, Brown P, Rowe BH, McAlister FA, Ezekowitz JA (2016). Insights into the importance of the electrocardiogram in patients with acute heart failure. Eur. J. Heart Fail..

[3] Rizwan A (2020). A review on the state of the art in atrial fibrillation detection enabled by machine learning. IEEE Rev. Biomed. Eng..

[4] Dupre, A., Vincent, S. & Iaizzo, P. A. Basic ecg theory, recordings, and interpretation. *Handbook of cardiac anatomy, physiology, and devices* 191–201 (2005).

[5] Hammad M, Pławiak P, Wang K, Acharya UR (2021). Resnet-attention model for human authentication using ecg signals. Expert. Syst..

[6] Singh P, Sharma A (2022). Attention-based convolutional denoising autoencoder for two-lead ecg denoising and arrhythmia classification. IEEE Trans. Instrum. Meas..

[7] Hou Y, Liu R, Shu M, Xie X, Chen C (2023). Deep neural network denoising model based on sparse representation algorithm for ecg signal. IEEE Trans. Instrum. Meas..

[8] Shan L (2022). Abnormal ecg detection based on an adversarial autoencoder. Front. Physiol..

[9] Wang J (2021). Automated ecg classification using a non-local convolutional block attention module. Comput. Methods Programs Biomed..

[10] Zhang J (2020). Ecg-based multi-class arrhythmia detection using spatio-temporal attention-based convolutional recurrent neural network. Artif. Intell. Med..

[11] Mousavi S, Afghah F, Acharya UR (2020). Han-ecg: An interpretable atrial fibrillation detection model using hierarchical attention networks. Comput. Biol. Med..

[12] Fan T (2023). A new deep convolutional neural network incorporating attentional mechanisms for ecg emotion recognition. Comput. Biol. Med..

[13] Vaswani, A. *et al.* Attention is all you need. *Advances in neural information processing systems* **30** (2017).

[14] Guo M-H (2021). Pct: Point cloud transformer. Computational Visual Media.

[15] Mark, R. & Moody, G. dbinfo.html. http://ecg.mit.edu/dbinfo.html. 1997, May.

[16] Snyder, C. & Vishwanath, S. Interpretable factorization for neural network ecg models. *arXiv preprint* arXiv:2006.15189 (2020).

[17] Hou, X., Qin, S. & Su, J. Two-stream network for ecg signal classification. *arXiv preprint* arXiv:2210.06293 (2022).

[18] Guo, C., Ahmed, S. & Alouini, M.-S. Machine learning-based automatic cardiovascular disease diagnosis using two ecg leads. *arXiv preprint* arXiv:2305.16055 (2023).

[19] Farhadi, J., Attarodi, G., Dabanloo, N.J., Mohandespoor, M. & Eslamizadeh, M. Classification of atrial fibrillation using stacked auto encoders neural networks. In: *2018 Computing in cardiology conference (CinC)*, vol. 45, 1–3 (IEEE, 2018).

[20] Odugoudar, A. & Walia, J.S. Ecg classification system for arrhythmia detection using convolutional neural networks. *arXiv preprint* arXiv:2303.03660 (2023).

[21] Ang, G.J.N. *et al.* A novel application for real-time arrhythmia detection using yolov8. *arXiv preprint *arXiv:2305.16727 (2023).

[22] Islam MS (2023). Hardc: A novel ecg-based heartbeat classification method to detect arrhythmia using hierarchical attention based dual structured rnn with dilated cnn. Neural Netw..

[23] Liu, C., Cheng, S., Ding, W. & Arcucci, R. Spectral cross-domain neural network with soft-adaptive threshold spectral enhancement. *arXiv preprint* arXiv:2301.10171 (2023). 10.1109/TNNLS.2023.3332217 37999966

[24] Alamatsaz, N. *et al.* A lightweight hybrid cnn-lstm model for ecg-based arrhythmia detection. *arXiv preprint* arXiv:2209.00988 (2022).

[25] Islam MR, Qaraqe M, Qaraqe K, Serpedin E (2024). Cat-net: Convolution, attention, and transformer based network for single-lead ecg arrhythmia classification. Biomed. Signal Process. Control.

[26] Akbar M, Nurmaini S, Partan RU (2024). The deep convolutional networks for the classification of multi-class arrhythmia. Bulletin of Electrical Engineering and Informatics.

[27] Zhang, F., Wang, J., Li, M. & Wang, B. Multi-scale and multi-channel information fusion for exercise electrocardiogram feature extraction and classification. *IEEE Access* (2024).

[28] Banerjee, D., Dey, S. & Pal, A. An snn based ecg classifier for wearable edge devices. In: *NeurIPS 2022 Workshop on Learning from Time Series for Health*.

[29] Wang M, Rahardja S, Fränti P, Rahardja S (2023). Single-lead ecg recordings modeling for end-to-end recognition of atrial fibrillation with dual-path rnn. Biomed. Signal Process. Control.

[30] Fayyazifar, N. An accurate cnn architecture for atrial fibrillation detection using neural architecture search. In *2020 28th European signal processing conference (EUSIPCO)*, 1135–1139 (IEEE, 2021).

[31] Chen Y (2018). Classification of short single-lead electrocardiograms (ecgs) for atrial fibrillation detection using piecewise linear spline and xgboost. Physiol. Meas..

[32] Zihlmann, M., Perekrestenko, D. & Tschannen, M. Convolutional recurrent neural networks for electrocardiogram classification. In: *2017 Computing in Cardiology (CinC)*, 1–4 (IEEE, 2017).

[33] Zhang, A., Yang, X., Li, T., Dou, M. & Yang, H. Classification method of ecg signals based on ranet. *Cardiovascular Engineering and Technology* 1–11 (2024). 10.1007/s13239-024-00730-5 38653933

[34] Xie J, Wang Z, Yu Z, Ding Y, Guo B (2024). Prototype learning for medical time series classification via human-machine collaboration. Sensors.

[35] Chen, Y. *et al.* Atrial fibrillation detection from compressed ecg measurements for wireless body sensor network. *ACM Transactions on Internet Technology* (2024).

